# Mitral Valve Atlas for Artificial Intelligence Predictions of MitraClip Intervention Outcomes

**DOI:** 10.3389/fcvm.2021.759675

**Published:** 2021-12-10

**Authors:** Yaghoub Dabiri, Jiang Yao, Vaikom S. Mahadevan, Daniel Gruber, Rima Arnaout, Wolfgang Gentzsch, Julius M. Guccione, Ghassan S. Kassab

**Affiliations:** ^1^3DT Holdings LLC, San Diego, CA, United States; ^2^Dassault Systemes Simulia Corp, Johnston, RI, United States; ^3^Department of Medicine, University of California, San Francisco, San Francisco, CA, United States; ^4^The UberCloud, Sunnyvale, CA, United States; ^5^Division of Cardiology, Department of Medicine, University of California, San Francisco, San Francisco, CA, United States; ^6^Bakar Computational Health Sciences Institute, University of California, San Francisco, San Francisco, CA, United States; ^7^Center for Intelligent Imaging, University of California, San Francisco, San Francisco, CA, United States; ^8^Biological and Medical Informatics, University of California, San Francisco, San Francisco, CA, United States; ^9^Chan Zuckerberg Biohub, University of California, San Francisco, San Francisco, CA, United States; ^10^Department of Surgery, University of California, San Francisco, San Francisco, CA, United States; ^11^Department of Medicine, California Medical Innovations Institute, San Diego, CA, United States

**Keywords:** mitral valve, finite element method, cardiac imaging, principal component analysis, MitraClip

## Abstract

Severe mitral regurgitation (MR) is a cardiac disease that can lead to fatal consequences. MitraClip (MC) intervention is a percutaneous procedure whereby the mitral valve (MV) leaflets are connected along the edge using MCs. The outcomes of the MC intervention are not known in advance, i.e., the outcomes are quite variable. Artificial intelligence (AI) can be used to guide the cardiologist in selecting optimal MC scenarios. In this study, we describe an atlas of shapes as well as different scenarios for MC implantation for such an AI analysis. We generated the MV geometrical data from three different sources. First, the patients' 3-dimensional echo images were used. The pixel data from six key points were obtained from three views of the echo images. Using PyGem, an open-source morphing library in Python, these coordinates were used to create the geometry by morphing a template geometry. Second, the dimensions of the MV, from the literature were used to create data. Third, we used machine learning methods, principal component analysis, and generative adversarial networks to generate more shapes. We used the finite element (FE) software ABAQUS to simulate smoothed particle hydrodynamics in different scenarios for MC intervention. The MR and stresses in the leaflets were post-processed. Our physics-based FE models simulated the outcomes of MC intervention for different scenarios. The MR and stresses in the leaflets were computed by the FE models for a single clip at different locations as well as two and three clips. Results from FE simulations showed that the location and number of MCs affect subsequent residual MR, and that leaflet stresses do not follow a simple pattern. Furthermore, FE models need several hours to provide the results, and they are not applicable for clinical usage where the predicted outcomes of MC therapy are needed in real-time. In this study, we generated the required dataset for the AI models which can provide the results in a matter of seconds.

## 1. Introduction

MitraClip (MR) is a percutaneous procedure to treat severe mitral regurgitation (MR), a disease with a prevalence of 4 million in US and an incidence of 250,000 patients each year (1, 2). This intervention is most appropriate for patients who cannot tolerate surgery because of other diseases or poor health conditions (2). The short-term and long-term benefits of MC has been reported in clinical studies (3–5).

Despite the benefits of MC implantation, the positioning of the clips is based on echocardiographic parameters including color doppler which does not accurately predict reduction of MR. Particularly, the location of the MC along the leaflet edges and the number of MCs has a direct role in the effectiveness of MC therapy. Currently, there is no systematic approach to determine the location and number of MCs that minimize MR. On the other hand, leaflet injury can occur as a result of MC intervention (6, 7), which is another consideration for an optimal MC therapy.

An *ad-hoc* approach to implant the MC in an optimal scenario would be to try different locations and number of MC, but this is not practical. Computational modeling including finite element (FE) analysis can be used to create *in-silico* models of MC procedure (8–11). Using FE, different scenarios can be virtually evaluated for a subject, and the best strategy is implemented during the MC intervention. FE computations are time-consuming, however, making FE models inappropriate for clinical usage. Artificial Intelligence (AI) and its subcategory Machine learning (ML) is being used in cardiovascular medicine and technology (12) and presents an opportunity to speed MC simulations compared to FE modeling.

To use AI for MC interventions, an atlas of shapes as well as different scenarios for MC implantation, namely the location and number of MCs is required. Notably, the dataset should cover the diversity in MV geometry for different subjects. The MV geometry can be acquired by different imaging modalities such as magnetic resonance imaging (MRI), CT scan, and echocardiography (Echo). Although Echo modality contains less details compared to MRI and CT scan, it is more convenient for patients. Since there are hand-held Echo imaging devices in development, this imaging modality is more likely to be used in future mobile technologies and internet of things (IoT) technology.

The aim of this paper is to introduce a methodology for creating an atlas that can be used for MV AI applications. Particularly, the data generation methods presented can be used to mine the MC intervention based on AI algorithms. Shapes and FE models of the MV are created where the geometries of the models are based on Echo images of the MV, the morphological data in the literature, principal component analysis (PCA), and generative adversarial networks (GANs). For each geometry, 7 scenarios were created whereby the MR in untreated MV, and 6 locations for the MC were simulated. We also show results for 2 and 3 MCs for comparison.

## 2. Methods

### 2.1. MV Geometry

To create the MV geometries, we morphed a template geometry using data from several key points. The key points were obtained from different sources as described below. The template MV geometry was adopted from another study (13). For morphing the geometry, PyGem, an open-source library in Python was used. We used Radial Basis Functions (RBF) in this package (14). We removed some chords from the original template (888 out of 4,971 total chordal elements). This was necessary to cause MR for some geometries obtained from normal valve data (as described below).

#### 2.1.1. Data From Patients

**Figure 1:**
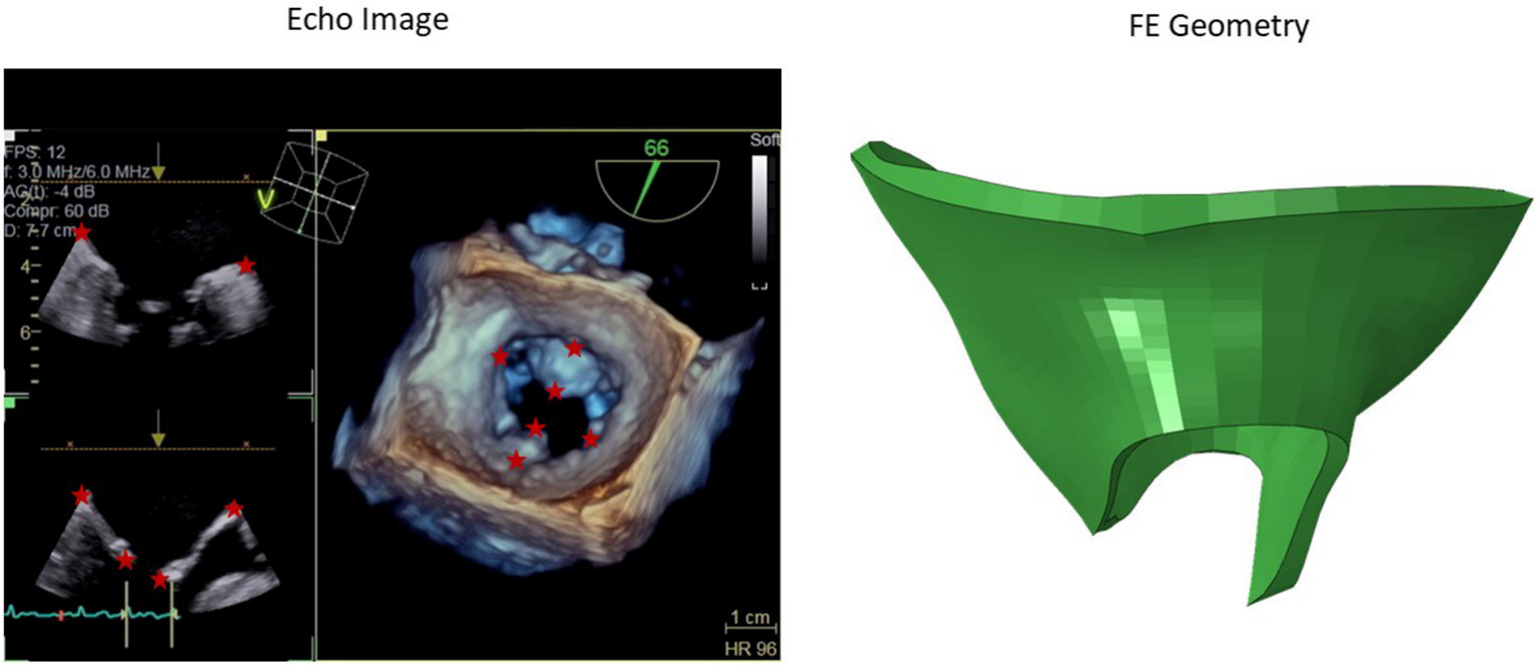
A sample 3D echo image. Six key points used in the morphing are shown with stars. The pixel data from these key points were obtained manually in ImageJ, and they were used to create the respective geometry by morphing a template geometry. The shape on the right is the respective patient geometry after morphing.

**Figure 2:**
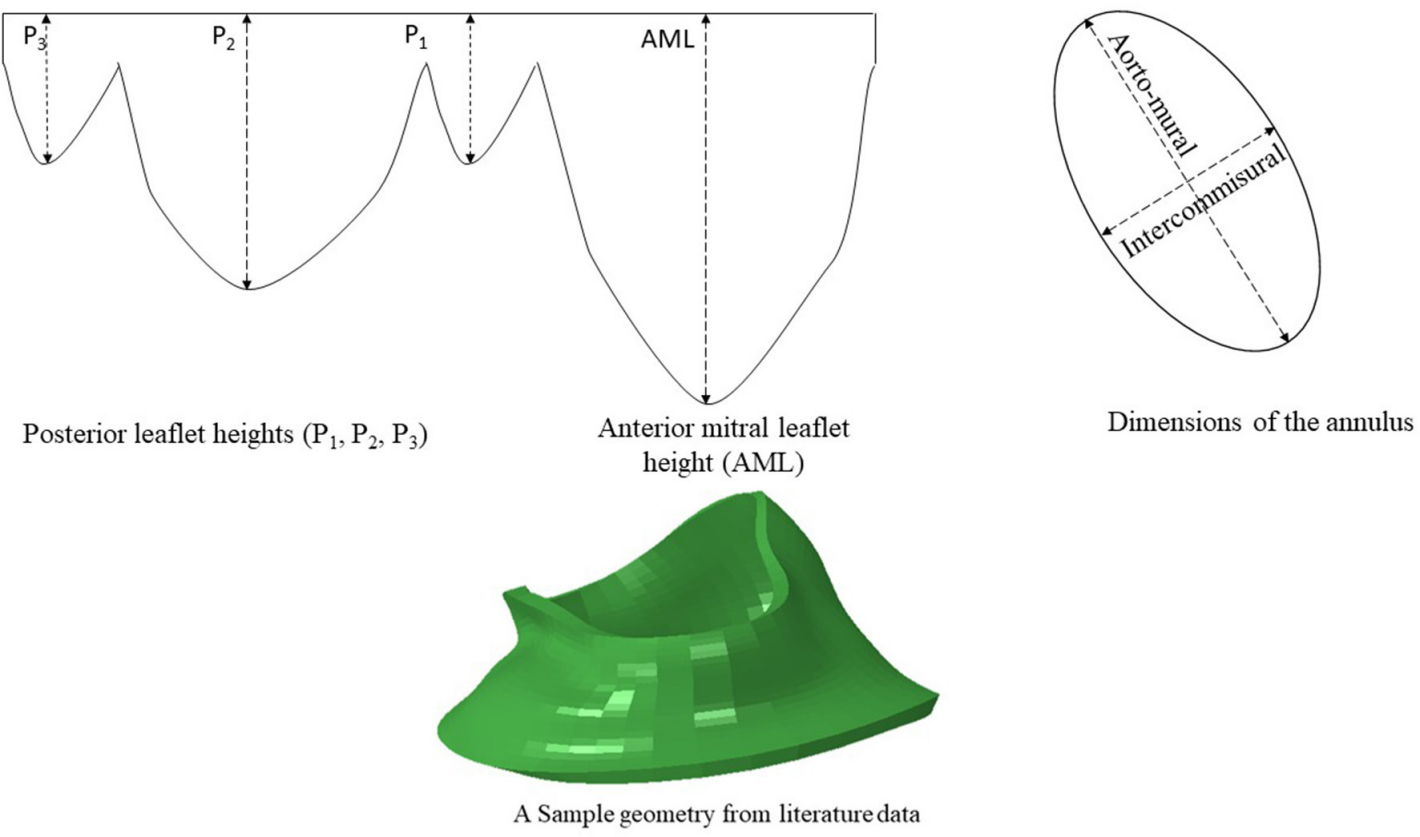
The dimensions of six key points were computed from literature. The diameters of the annulus (two dimensions) and the leaflet edge dimensions (four points) were adopted from data in the literature (15). A sample geometry created from literature data is shown.

We obtained 3-dimensional (3D) echocardiographic image data from University of California San Francisco (UCSF). The images obtained were in accordance with UCSF Institutional Review Board (number 19-27738). We used Echo images from UCSF to create a portion of the dataset. An example Echo image is shown in Figure 1. We used ImageJ software (version 1.53e, National Institutes of Health) to manually obtain the pixel coordinates of several key points (Figure 1) in each image. The pixel data obtained in three views from the images, were used to morph the template geometry (Figure 2). In total, we reconstructed geometries from 29 patients.

It should be noted that the MV geometries pertain to patients that were approved for MC intervention at UCSF Medical Center. Thus, the geometries obtained from these images represent pathological MVs that require MC intervention.

#### 2.1.2. Data From Literature

Another source for creating the geometry is from data in the literature. We used the statistics provided by Krawczyk-Ozóg et al. for the leaflets [Figure 2; (15)]. A normal distribution for the leaflet parameters was used in our study. Using the mean and standard deviation from the dataset provided by (15), we calculated the respective data for 5, 50, 95% percentile of population. The template geometry was morphed using the data for each datapoint to create respective geometry. The resulting values are shown in Table 1. For each of the aorto-mural and inter-commissural diameters, five values were used while other morphological parameters were fixed (Table 1).

**Table 1:** The values used for the leaflet morphological parameters.

**Parameter**	**Values (mm)**
AML	13.63, 20.6, 27.57
P_1_	6.85, 11, 15.15
P_2_	8.25, 12.9, 17.55
P_3_	6.08, 10.4, 14.72
Aorto-mural diameter	21.5, 22.0, 22.5, 23.0, 23.5
Intercommissural diameter	17.65, 18.15, 18.65, 19.15, 19.65

*For the variations in diameters, other parameters were fixed at following values: AML = 13.63 mm, P_1_ = 6.85 mm, P_2_ = 8.25 mm, P_3_ = 6.08 mm*.

This part of the dataset represents normal MV morphologies. MV geometries that require MC could have characteristics that are not seen in normal MV (such as the length of leaflets and annulus diameter). In some patients with MR, however, the MV morphology may be normal (16, 17). Unlike imaging data, the geometries created from these data source were not directly obtained from patients. Rather, they were used for data augmentation.

#### 2.1.3. Data From PCA

We used PCA to generate more virtual patients' data using the data we had. The coordinates from UCSF patients' data and data from literature were used for this purpose. We used the following equation for this purpose:


(1) $$\begin{array}{r} X≅X_{PCA}=\bar{X}+\overset{M}{\underset{m=1}{\sum }}\alpha_{m}\sqrt{\lambda_{m}}W_{m} \end{array}$$

where $\bar{X}$ is the mean shape, and {*W*_*m*_} and {λ_*m*_} are the eigenvectors and eigen values of the covariance matrix, respectively, {α_*m*_} is the shape code, and *M* is number of principal components (18–20). We used three components as they could describe over 90% of total shape variation (21). We used linear algebra library in Python to apply singular value decomposition on the MV dataset, and then, PCA computations followed. In total, the valve dataset used for PCA contained 180 shapes.

The geometries obtained from PCA were a combination of MVs from patients and normal MVs. Similar to the geometries created from literature (section Data From Literature), the geometries obtained from PCA were used for data augmentation.

#### 2.1.4. Data From GANs

After creating the dataset from image dataset and literature, we had a dataset from different geometries. Using this dataset, we generated more images using GANs (22). This algorithm has two parts. One part generates the geometries (the generator) and the other part tries to distinguish the generated geometry from a real geometry (discriminator). Initially, the discriminator can recognize the generated image from the real image, but as the generator trains, the discriminator has difficulty in separating the generated image from the real images. To implement this algorithm, we treated the geometries as images. A deep learning (DL) model in TensorFlow was adopted for this purpose (23). We used TensorFlow for computations in Google Collaboratory with Graphics Processing Units (GPUs), with following specifications: name: Tesla P100-PCIE-16GB, driver version: 460.32.03, memory: 16280 MiB.

### 2.2. Computational Set Up

**Figure 3:**
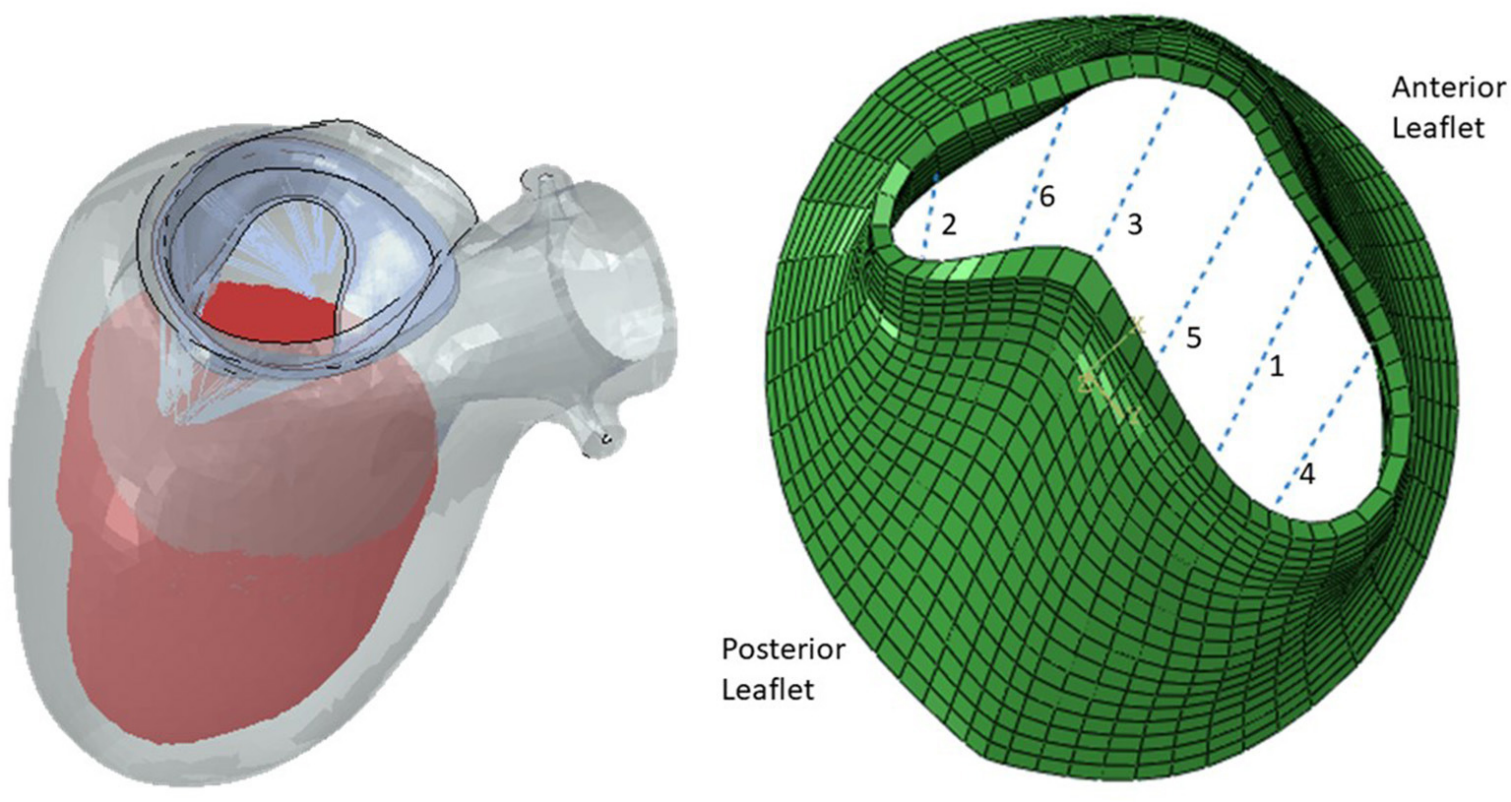
The FE model **(left)** and the MV **(right)**. The six locations for MC are shown.

After the geometries for the MVs were developed, they were used to conduct FE simulations. The FE model included the MV, its chords and the left ventricle (LV), as indicated in Figure 1. As mentioned above, the MV geometry was adopted from another study (13). The blood flow was modeled using Smoothed Particle Hydrodynamics (SPH) where particles represent the red blood cells. The MC was simulated by connecting respective points on the MV leaflet edges (Figure 3).

The leaflet materials were modeled using hyperplastic fiber-reinforced material, as below (24, 25):


(2) $$\begin{array}{r} {\text{ Ψ }}_{dev}=\frac{a}{2b}e^{b\left(I_{1}-3\right)}+\overset{}{\underset{i=f,t}{\sum }}\frac{a_{i}}{2b_{i}}\left\{e^{b_{i}{\left(I_{4i}-1\right)}^{2}}-1\right\}\\ \;+\frac{a_{ft}}{2b_{ft}}\left\{e^{b_{ft}{\left(I_{8ft}\right)}^{2}}-1\right\}\\ {\text{ Ψ }}_{vol}=\;\frac{1}{D}\left(\frac{J^{2}-1}{2}-\ln\;\left(J\right)\right) \end{array}$$

where *a* and *b* are isotropic stiffness of the tissue; *a*_*f*_ and *b*_*f*_ are tissue stiffness in the fiber direction, *a*_*t*_ and *b*_*t*_ are tissue stiffness in the (transverse) plane perpendicular to the fiber direction, *and a*_*ft*_ and *b*_*ft*_ are shear rigidity between fibers and transverse plane. *I*_1_, *I*_4*i*_ and *I*_8*ft*_ are invariants, as follows:


(3) $$\begin{array}{l} {\text{I}}_{1}:=\text{tr}\left(\text{C}\right)\\ {\text{I}}_{4i}:=\text{C}:\left({\text{f}}_{0}⊗{\text{f}}_{0}\right)\\ {\text{I}}_{8fs}:=\text{C}:\text{sym}\left({\text{f}}_{0}⊗t_{0}\right) \end{array}$$

**C** is the right Cauchy-Green tensor, **f**_0_ and **s**_0_ are vectors that define the fiber and trans-fiber directions, respectively. *J* is the deformation gradient invariant and *D* is a multiple of the Bulk Modulus *K* ($\frac{2}{K}$).

We used LV (including papillary muscles) as the surrounding geometry for the blood flow and did not consider deformations in the LV nodes. The MV annulus changed for different geometries, but the LV geometry was the same. This caused a mismatch between LV and MV annulus. To avoid leakage between LV and MV due to this mismatch, a surface was placed to seal the gap. This surface did not affect the deformations of the MV and only sealed the gap between MV and LV. The blood flow was enforced by a plate in the LV side of the MV that moved toward the MV. A pressure was applied to the leaflets to close them. The MV annulus motions were adopted from the literature. The SPH methodology is similar to previous publications (24, 26).

The FE simulations were conducted in several steps: (1) SPH particles entered the LV and subsequently the model replicated an LV filled with blood; (2) MC was implemented by connecting respective nodes from two leaflets; (3) Pressure load was applied to close the leaflets; and (4) Particles were forced to move toward the MV, using a plate.

The FE software Abaqus Unified FEA (Part of 3DEXPERIENCE SIMULIA software Suite, Dassault Systemes, Providence, RI, USA) version 2020 was used for computations. For leaflets, we used a material available in Abaqus Living Heart license that is based on Equation 2. The leaflets element type was C3D8I (8-node linear brick, incompatible modes); the chords element type was T3D2 (linear 3-D truss), and the blood particles element type was C3D8R (8-node linear brick, reduced integration with hourglass control). The Abaqus explicit solver was used with automatic time incrementation, and mass scaling was used. The general contact (including self-contact) was used for interaction between the model components including blood particles, leaflets, LV, and the plate which was used to move the particles toward atrium. The FE computations were conducted on the UberCloud platform using Google Cloud Platform computational nodes. We used C2 instances (Cascade Lake) with 30 cores and 240 GB of main memory for computations. The average runtime for each FE model was 6 h.

## 3. Results

**Figure 4:**
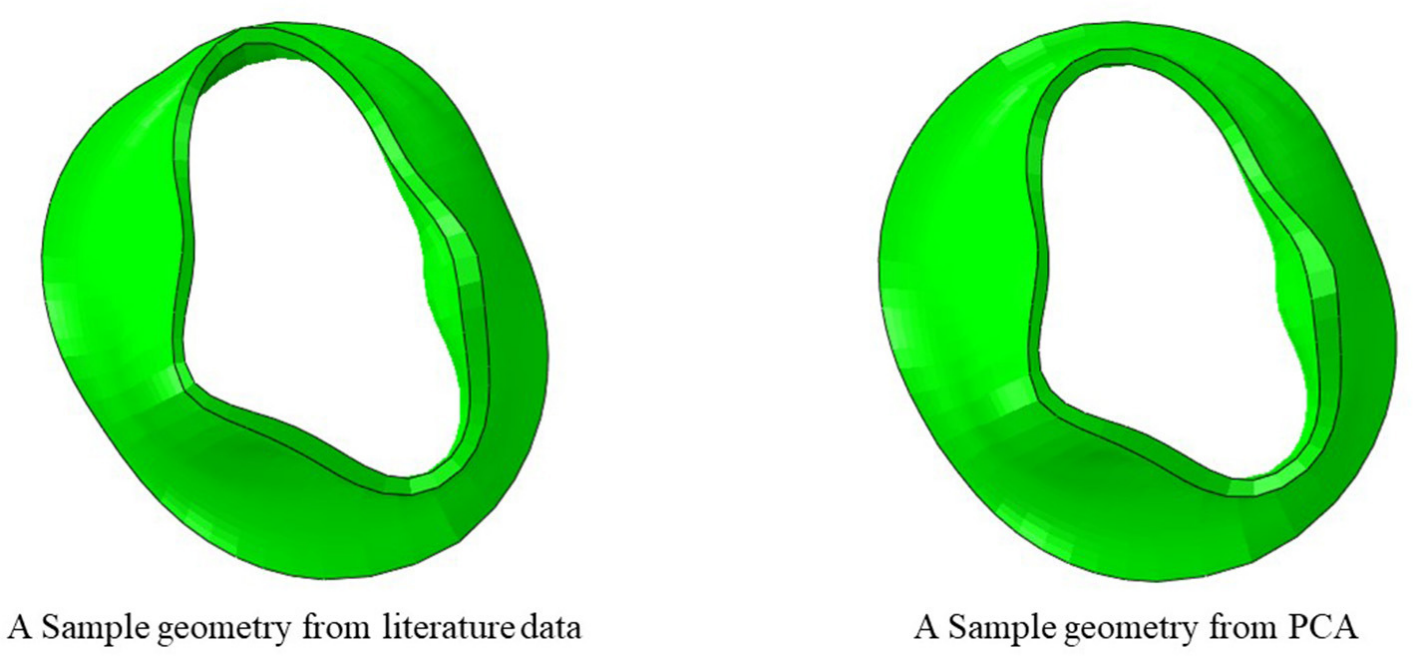
A model created from literature data and a model created using PCA.

**Figure 5:**
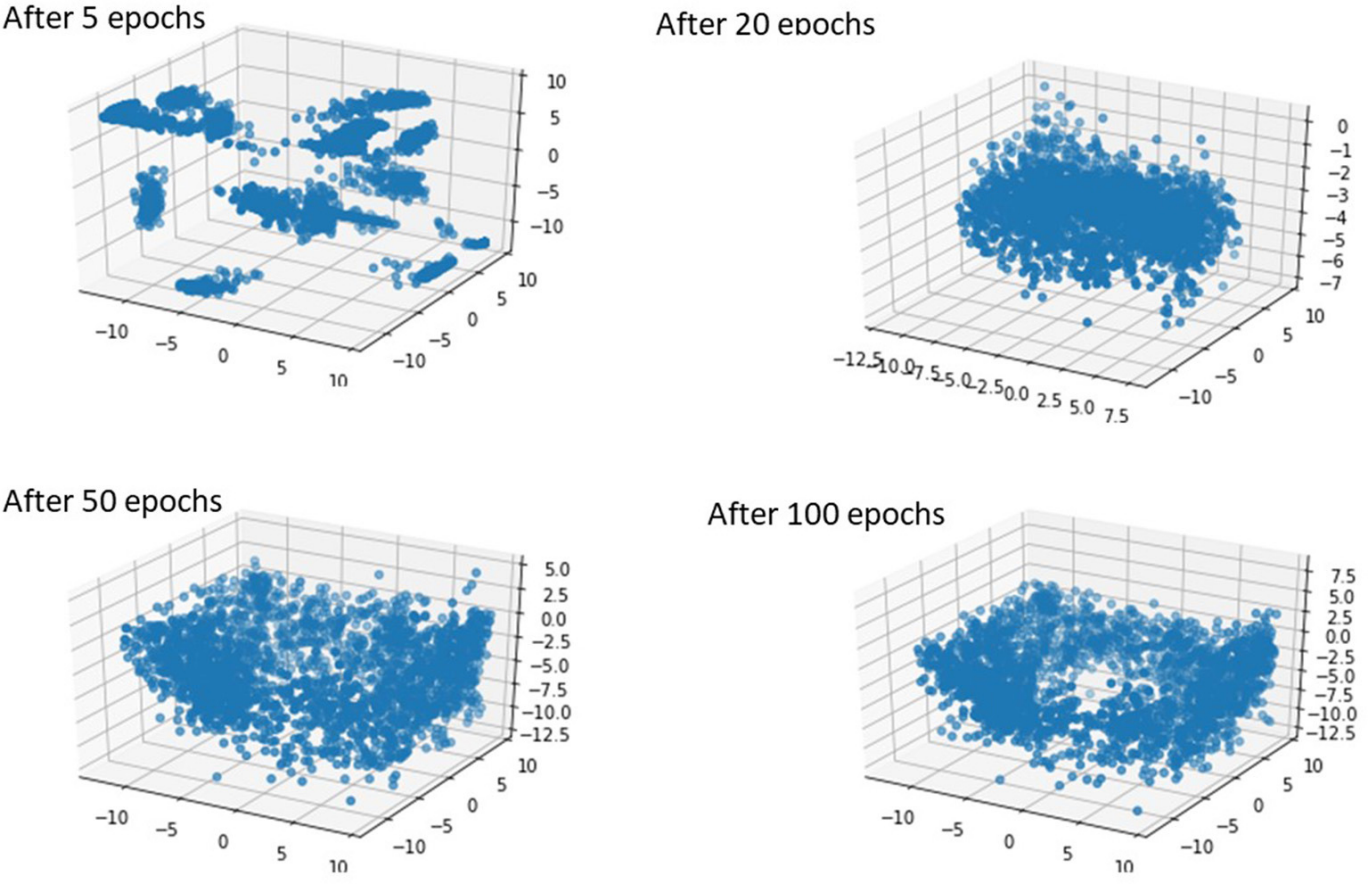
The GAN model was able to produce MV geometries after several epochs.

**Figure 6:**
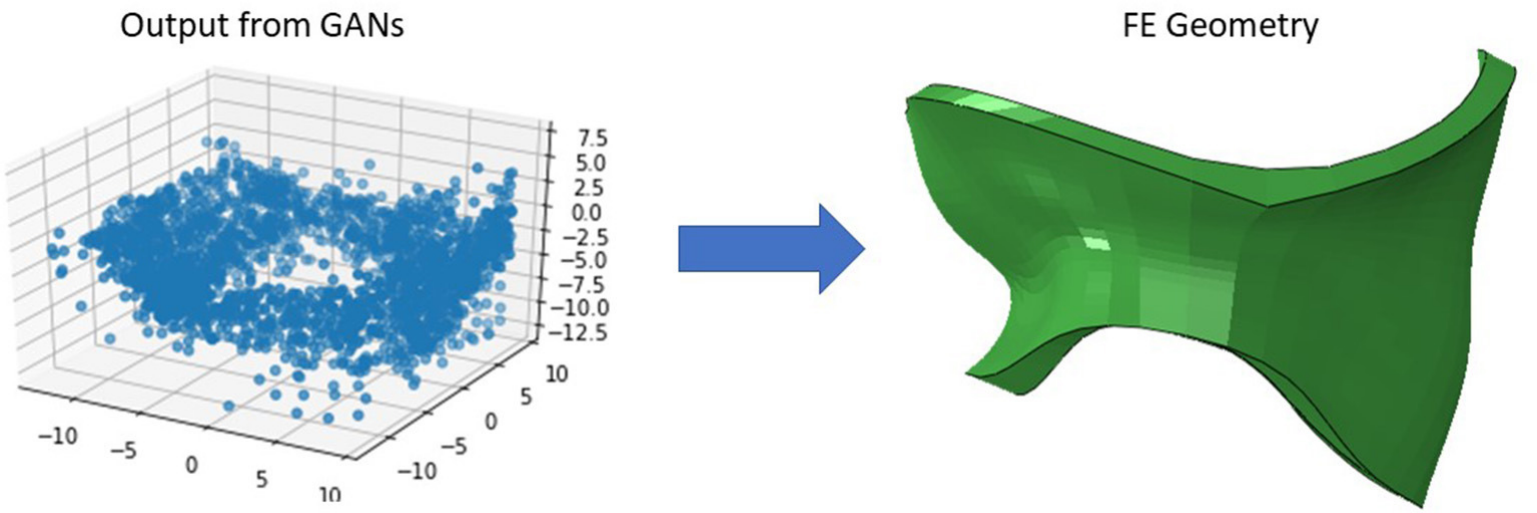
The GAN-generated geometries became more similar to real MVs after several epochs.

We created 55 geometries from image data, 106 geometries based on leaflet parameters in the literature, and 20 geometries using PCA (Figure 4). Although GANs could generate additional shapes (Figures 5, 6), we did not use it to create more geometries, as it required more computational costs. The GANs algorithm required several epochs to generate MV models that were similar to the dataset (Figure 5). Although in the initial epochs the geometries provided by GANs were randomly distributed, the generated geometries became closer to the MV (Figure 5). The output from GANs was not directly useable for FE modeling, but it provided coordinates of 6 key points which could be used to morph the template FE model (Figure 6). The result shown in Figure 6 was obtained from 81 geometries based on geometrical parameters from the literature.

**Figure 7:**
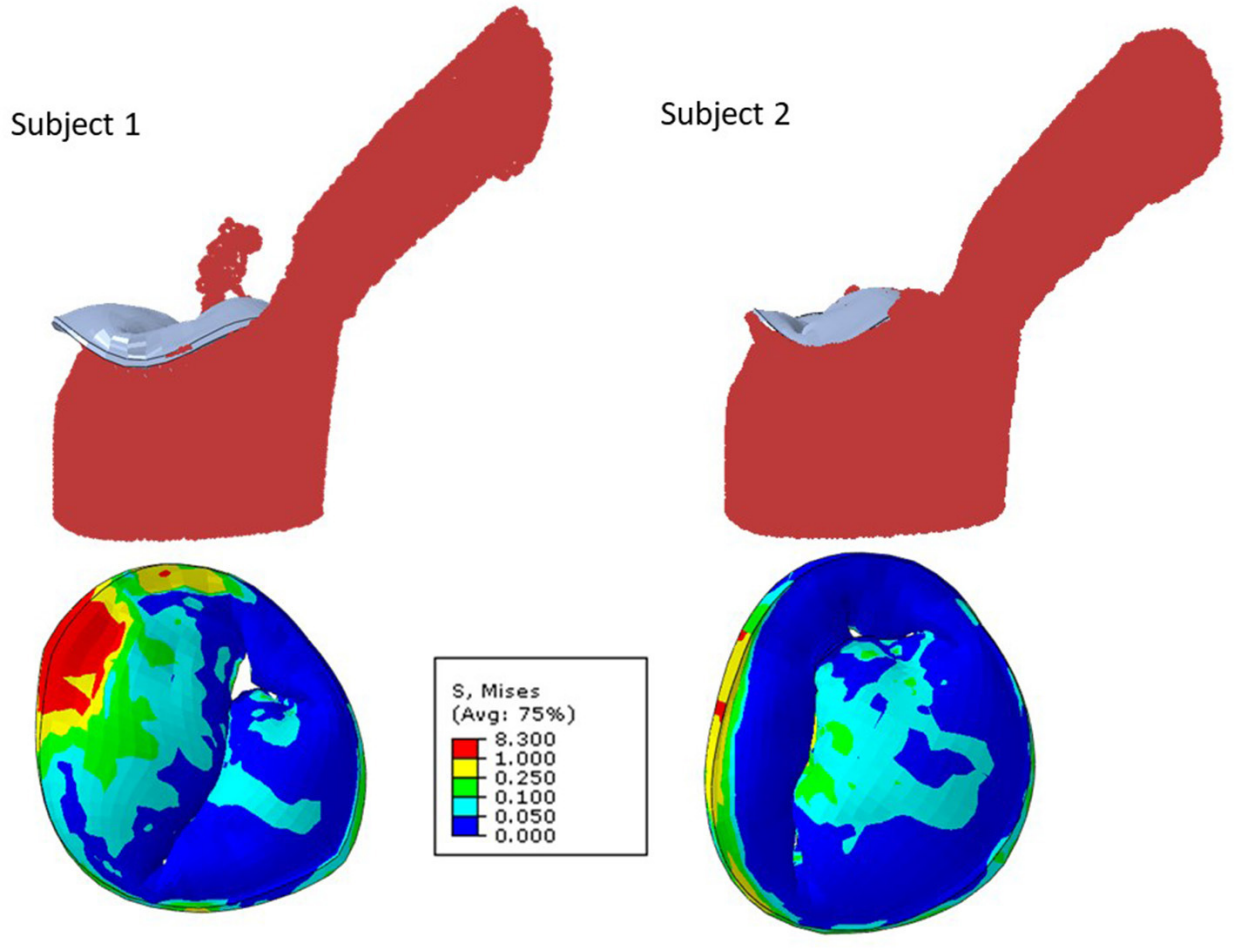
The MR and leaflet stress for two different subjects.

**Figure 8:**
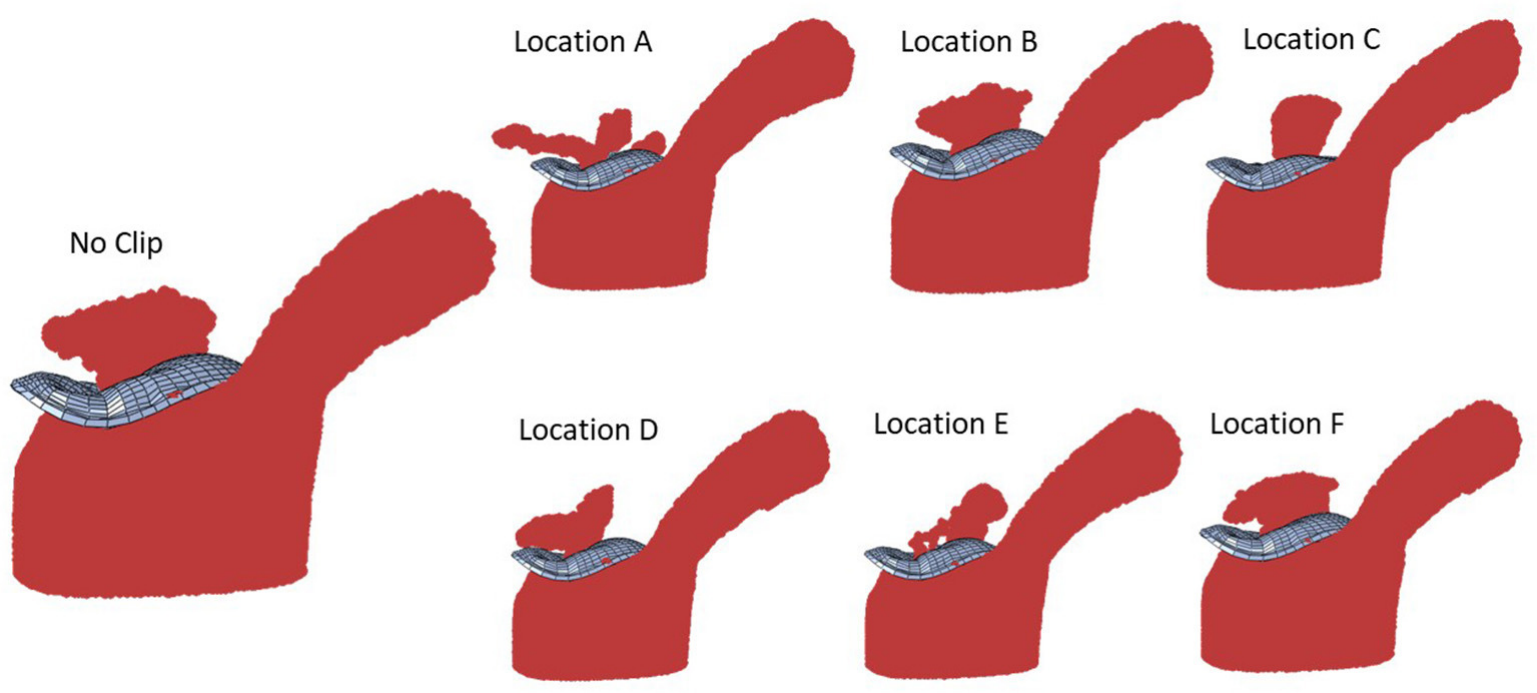
The MR for “No Clip” and 6 locations for a single MC (the same geometry).

**Figure 9:**
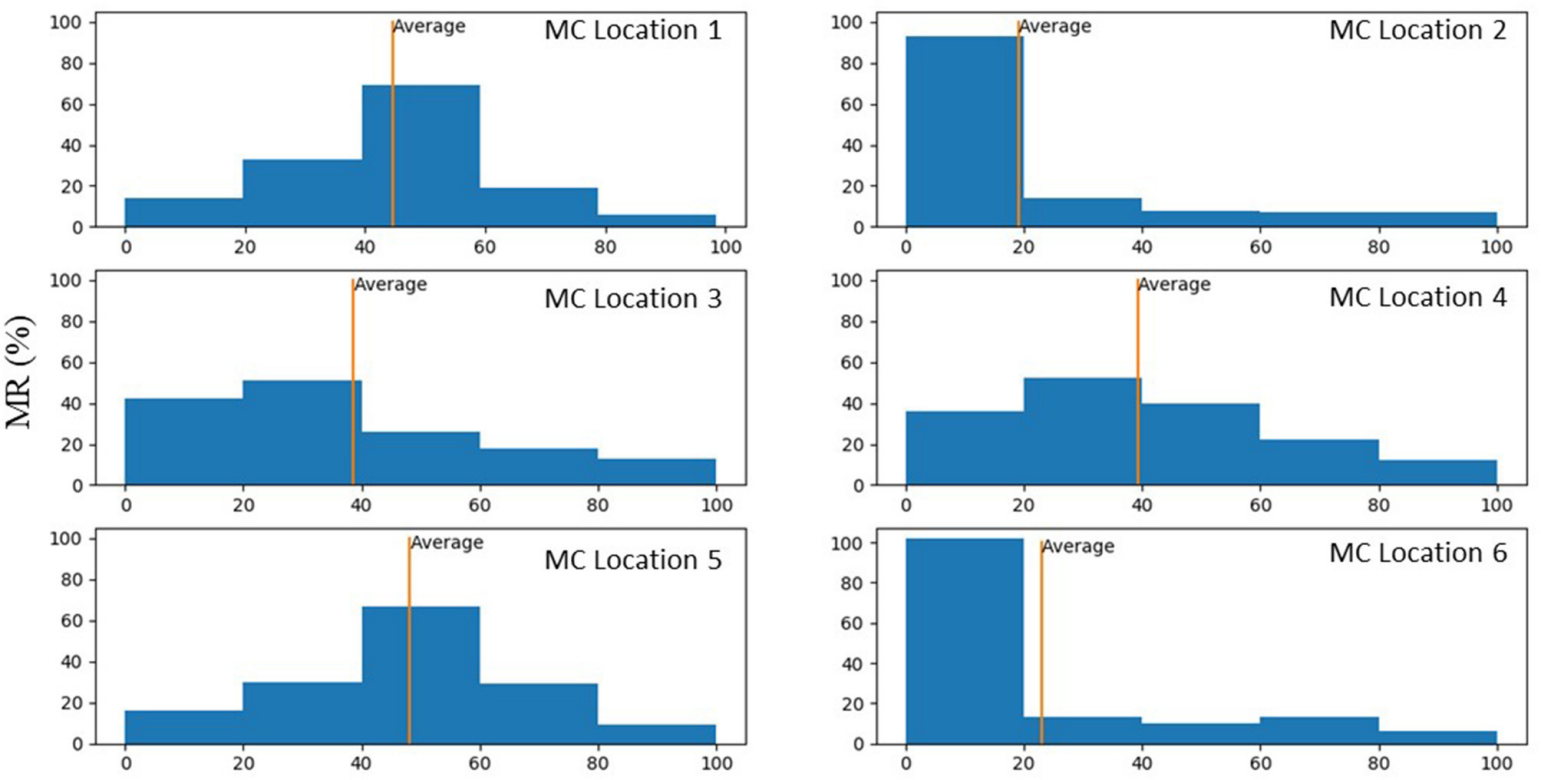
Histograms that show percentage of MR reduction in the MV for each MC location. These histograms were plotted only for scenarios where MR reduced after MC intervention.

**Figure 10:**
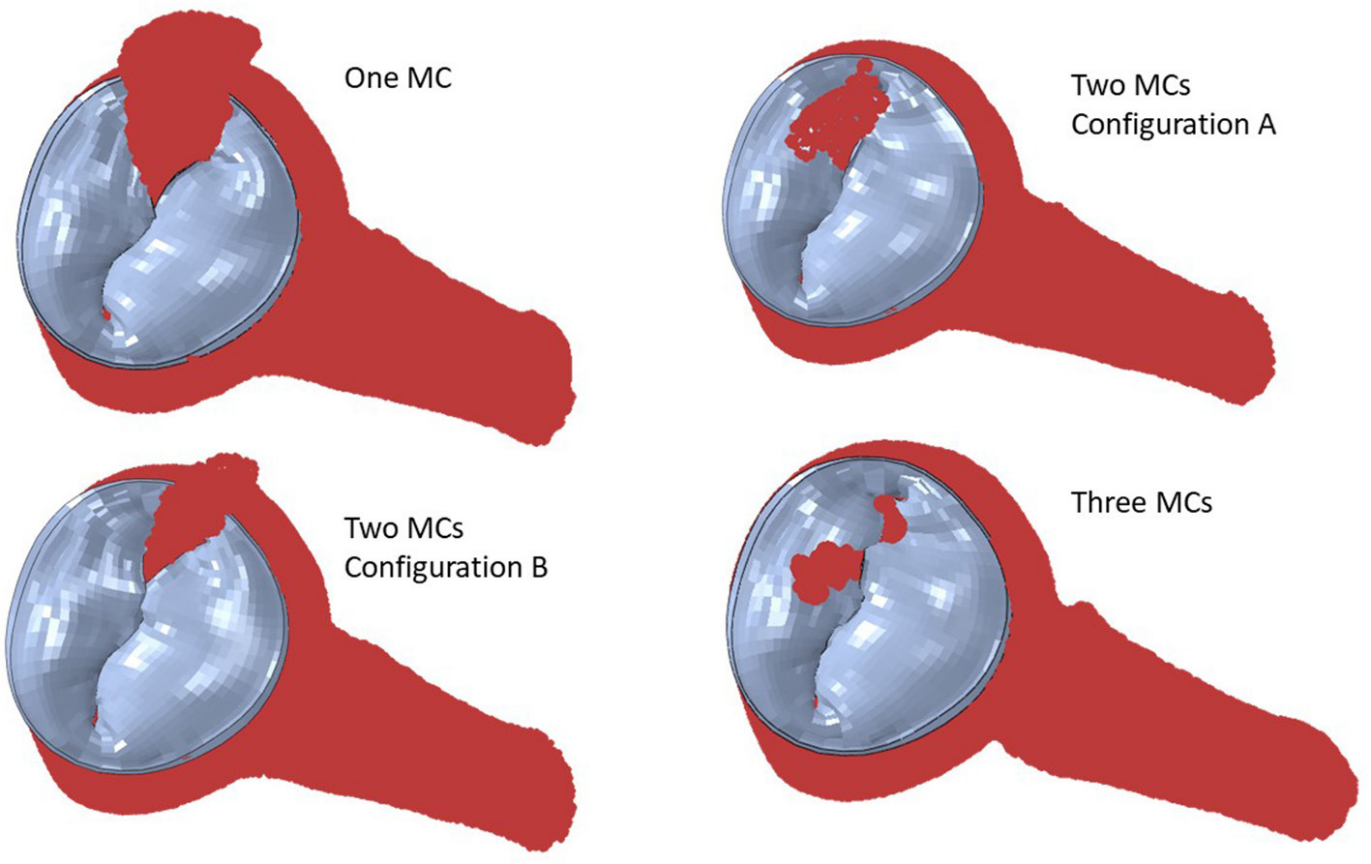
The MR for two and three MCs. For two clips, **(A)** represents clips at locations 3 and 4, and **(B)** represents clips at locations 3 and 5.

The blood flow through the MV was simulated by the FE models. The MR was different for different geometries (Figures 7–**10**). As well, other parameters were affected including the deformations in the leaflets and stress distributions.

With one MC, with different MC locations, the MR was altered, and the alterations in MR was more noticeable for some MC locations (Figures 8, 9). Other parameters, including the stress distributions and leaflet deformations changes with MC location.

**Figure 11:**
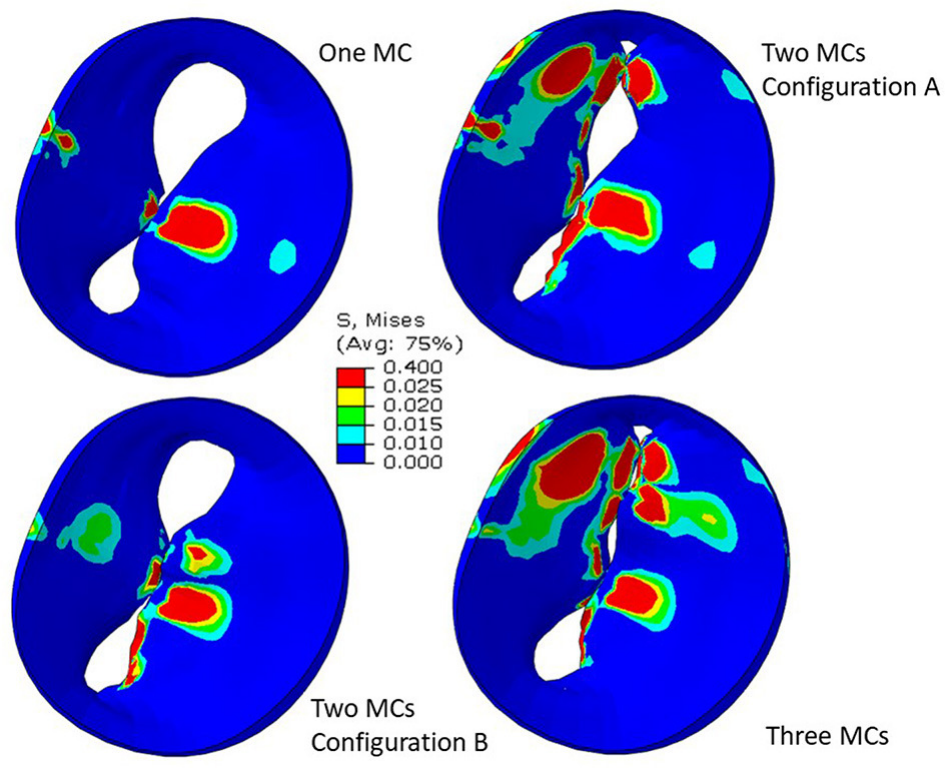
Leaflet von Mises stresses for one, two, and three MCs. These figures show the implantation of the MC. For two clips, **(A)** represents clips at locations 3 and 4, and **(B)** represents clips at locations 3 and 5.

Simulation scenarios with two and three MCs showed the MR with more than one clip. More than one MC led to lower MR compared to one MC, but the reduction in MR depended on the locations of the MCs. When two MCs are used, the locations of the two MCs with respect to each other, affects MR and stresses. The results for three MCs could lower the MR compared to two MCs. The stresses in the leaflets were altered with three MCs (Figures 10, 11). The number of MCs noticeably affected the orifice area. As the number of MCs increased, the MV orifice area became smaller (Figure 11).

## 4. Discussion

We presented a methodology to generate an atlas for prediction of MC intervention outcomes. Our approach is based on different data-generation methods, namely patients' data, literature reports, PCA, and GANs. In case the data from patients is limited, our approach can be used to generate/augment the data. We used echo images as one source of data generation as this modality is more available and safer than other modalities, and it is less expensive. Our FE modeling simulated fluid-structure interaction (FSI) for different scenarios for MC interventions.

The patient data that we used are based on Echo images. There are different modalities for MV imaging including MRI and CT. We used Echo modality because this imaging method is more readily available, less costly and safer. There are patients who cannot tolerate MRI; e.g., those with pacemakers. On the other hand, hand-held Echo cardiac imaging devices are progressing to the clinics (27). Therefore, our approach is compatible with current efforts for personalized imaging methods. These technologies that are integrated in mobile devices will expand Internet of Things (IoT) technologies, providing patient-oriented medicine and more effective treatments. On the other hand, working with Echo images is more complicated than MRI. The Echo images do not come in a regular DICOM format that can be directly used for geometrical reconstruction. Some vendors have provided software modules that makes it possible to convert DICOM data to 3D Cartesian format (Philips), but particularly, Siemens machines do not provide such a module. Our methodology contributes to using Echo images for MV simulations.

The outputs of PCA were directly appropriate for FE modeling (Figure 4). The GANs could provide a tool for MV data augmentation. In comparison to PCA, GANs were recently introduced (22). This deep learning algorithm has been used in many domains, particularly for computer vision. The outputs of GANs were not directly appropriate for FE modeling, but they provide the information to generate FE models; e.g., coordinates of key points that we used for morphing. Given that GANs provided the shapes after several epochs (Figures 5, 6), we used PCA for the data generation in this study.

Our study emphasizes using ML analysis for MC intervention optimization. First, it is not feasible to experiment with MC location to determine the optimal MC implantation strategy. Furthermore, stresses in the leaflets cannot be measured experimentally. The FE modeling can be used to virtually simulate the outcomes of different MC intervention scenarios. FE modeling (especially when FSI is considered), however, can be challenging. For some patients, the geometry of the leaflets deviates from a normal geometry such that the distortions in FEs cause numerical instability. This implies that FE modeling is limited in terms of numerical convergence for some patients. Moreover, FE modeling is time-consuming especially when FSI is involved. Even after FE computations are complete, they do not directly lead us to MC intervention improvements. Notably, based on the results, for the same MV geometry the alterations in MR does not follow a pattern that can be intuitively extracted from the data (Figure 9). Similar to MR, results for leaflet stresses also do not show a pattern by a simple analysis. In other words, the interplay between factors that affect MR and leaflet stress is complicated, making it difficult to provide a rule of thumb for optimal MC intervention.

On the other hand, ML models do not suffer from FE modeling limitations mentioned above. Once a ML model is developed, it can provide the results in much shorter time than FE modeling (25, 28), and it can provide the results for the MV parameters that are within the data distribution (results will be provided except for outliers). In this paper, we described data generation, an important step in using ML analysis for MC outcomes prediction.

Because our data generation is founded on FE models, we aimed to use a FE modeling approach that captures import information about MV dynamics and at the same time is computationally efficient. Our FE model has two important aspects that makes it more advanced compared to previous studies. First, it includes the LV as the surrounding geometry for blood whereas in our previous report, we used a cylinder (26). Because FSI models are relatively complex, inclusion of LV is important from a FE modeling standpoint. Second, our FE modeling approach is adaptable for different parameters of the MV, including geometrical parameters. This aspect is important because FSI models are typically sensitive to variations in model parameters. Moreover, we modeled not only the MV and LV but also the MC and different scenarios for MC implantations were considered. The average runtime for our FE models made it possible to use them for our data generation workflow (~6 h). We are not aware of a previous study that provided a workflow to create a dataset of MV FSI models for different scenarios of the MC intervention.

The MR and leaflet stress are important parameters for MC intervention. In particular, the locations and number of MCs are crucial factors for the outcome of the intervention. Our simulations show that when one MC is used, the stress and MR is different for each MC location (Figure 8). Similarly, when the number of MCs increased to 2 and 3, the MR and stresses were altered (Figures 10, 11). For the model shown in Figures 10, 11, with one clip (location 3), the reduction in MR was 40%, with two clips it was 83% (A) and 61% (B), and with three clips it was 95%. The maximum von Mises stress in the leaflets for one clip was 15.1 MPa, for two clip it was 13.2 MPa (A) and 11.2 MPa (B), and with three clips it was 17.0 MPa. Moreover, the geometry of the MV is another factor that plays a role in the outcomes of MC intervention (Figure 7). The maximum von Mises stress was 8.3 and 2.2 MPa for subjects A and B, respectively, in Figure 7.

Another important aspect of MC intervention is the alterations in the orifice area caused by MC(s) (29). Based on patient's background and preexisting pressure gradients, typically two or more clips run the risk of causing mitral stenosis (30). Our FE models simulate the effects of MCs on the orifice area as number of MCs change (Figure 11). Specifically, for the geometry in Figure 11, in relation to one clip scenario, two clips reduced the orifice area by 54% (A) and 23% (B). With respect to one clip, however, three clips reduced the orifice area by 72%. Therefore, our results also show that 3 MCs have noticeably higher effects on the MV orifice area.

The average MR reduction obtained by implanting a MC in locations 1 (45%), 3 (39%), 4 (39%), and 5 (48%) were relatively similar to each other. The average MR reductions obtained by implanting a MC at locations 2 (19%) and 6 (23%), however, were noticeably lower (Figure 9). The histograms for MR reductions for locations 2 and 6 are relatively similar. The similarity between MR reduction for locations 2 and 6 can be explained based on the vicinity between these two locations, and that they are both closer to the posterior commissure than the anterior commissure. Figure 9 demonstrates that the average values of MR should be interpreted with caution. Notably, for some geometries, a MC implanted in location 2 or 6 led to a larger reduction in MR than other locations. As such, it is important to determine the optimal MC scenarios based on each patient's parameters rather than average data.

## 5. Limitations

The MV has a wide range of parameters that can be different for each patient. These parameters include (but are not limited to) the geometrical specifications of the annulus and leaflets, the chords, the material properties, and the functional/degenerative type of the disease. The methodology we present in this paper is a proof of concept study that can be used for different types of MV diseases. Once the dimensions of the MV for a patient is extracted from the respective images, they can be used to drive the FE geometry using the morphing process explained in “Methods” section. As mentioned above, FE models do not converge for all geometries, but ML models can provide the results for that patient as long as the geometry distribution is within the dataset distribution range. As such, for a data set generation methodology, it is important to include data with a wide range of variance, as much as information is available.

We presented several methods to create the MV geometries including echo images, morphological data (from literature), PCA, and GANs. Each method provides a different approach to generate data. As such, the proposed methodology can utilize information in the form of either images or MV dimensions to create the dataset and utilize PCA and GANs to augment the data. In this study, the size of available images was limited. As such, morphological data from normal MVs were used to augment the data. This is a limitation in terms of data distribution because normal MV morphologies can be different from patients' morphologies. This limitation related to the available data, can be addresses as we collect more patient images or morphological data from pathological MVs (that need MC) becomes available. It should be noted that MR can be caused by chordae or papillary muscle rupture (17), in which case the MV morphology might be normal. Therefore, a comprehensive dataset for MC intervention needs to include MVs with normal morphology.

For ML simulations, a larger training data set is generally more desirable. Because the echo images provide the specifications of the patients' geometries directly applicable to MC therapy, there is a need to collect more images from a wide variety of patients. A larger patient images dataset can improve the distribution of geometries and the size of the training dataset. The former will cover more patients' geometries and the latter will improve the ML model accuracy. We are collecting more MV images from patients that need MC intervention.

We did not use GANs for creating additional parts for our dataset in this paper, but we showed that GANs are trainable for generating MV geometries. This result is important because PCA and GANs are two different approaches. The former finds pattern in data by looking at directions of largest orthogonal variances, but the latter finds a complex non-linear function between the inputs and outputs. Therefore, GANs can provide virtual geometries that are not created by PCA. In our future work, GANs can be used to augment geometries from patient data.

An additional limitation is that the LV was considered as the surrounding geometry for the blood flow simulations, which did not consider deformations of the LV; i.e., the LV geometry remained unchanged. In our simulations, we found the FSI convergence is sensitive to the model geometrical specifications. A model that uses a rigid LV geometry is more likely to encounter convergence failure than a model that uses a rigid cylinder. Thus, we included a rigid LV geometry since it is a step closer to a more evolved MV model. Future models require FSI that involves both MV and LV. The LV deformations should be considered in future models where the contraction of the LV will cause the ejection of the blood. This future work can integrate our LV models (25, 28) with our MV and MC modeling approach. The MV will be a part of the LV in such a way that the plate used to seal the gap between MV and LV will not be required. Also, annulus deformations will be caused by LV deformations, and the motions at the annulus will not be applied as boundary conditions. It should be noted that such a model likely requires tremendous computational resources, particularly if it is used for data generation.

Since the effects of MC on MR and MV dynamics do not follow simple patterns or rules, an AI tool is needed for such an analysis. This paper described a methodology to generate the dataset for AI-based tools for MC intervention improvements, but we did not present an AI tool. The next step in the AI analysis is to apply AI models on the data. Analysis of the data and applying AI models for prediction of MC outcomes can be topics of future publications.

## Data Availability Statement

The datasets presented in this article are not readily available because of limitations in data access policy in corresponding institutes. Requests to access the datasets should be directed to gkassab@calmi2.org.

## Author Contributions

YD, JG, and GK designed the study. YD conducted the simulations and analyzed the results. JY contributed to ABAQUS model development. DG and WG contributed to running the models. VM and RA contributed to data preparation and analysis. All authors read and revised the manuscript.

## Funding

This work was supported in part by the SBIR grant number R43 HL145896.

## Conflict of Interest

YD is an employee of 3DT Holdings LLC. JG is a consultant for, and JY is an employee of, Dassault Systèmes Simulia Corporation (Johnston, RI, USA). VM is PI for clinical research with Abbott, Edwards Life Sciences and GORE. WG and DG are employees of the UberCloud. The remaining authors declare that the research was conducted in the absence of any commercial or financial relationships that could be construed as a potential conflict of interest.

## Publisher's Note

All claims expressed in this article are solely those of the authors and do not necessarily represent those of their affiliated organizations, or those of the publisher, the editors and the reviewers. Any product that may be evaluated in this article, or claim that may be made by its manufacturer, is not guaranteed or endorsed by the publisher.
